# Neonatal near miss: a systematic review

**DOI:** 10.1186/s12884-015-0758-y

**Published:** 2015-12-01

**Authors:** Juliana P. Santos, Cynthia Pileggi-Castro, Jose S. Camelo, Antonio A. Silva, Pablo Duran, Suzanne J. Serruya, Jose G. Cecatti

**Affiliations:** Department of Gynecology and Obstetrics, School of Medicine, University of Campinas (UNICAMP), Campinas, Brazil; Department of Pediatrics, Ribeirão Preto Medical School, University of São Paulo (USP), Ribeirão Preto, Brazil; Department of Public Health, Federal University of Maranhão, Sao Luis, Brazil; Latin American Center of Perinatology (CLAP), Pan-American Health Organization (PAHO), Montevideo, Uruguay

**Keywords:** Neonatal morbidity, Neonatal mortality, Neonatal near miss, Systematic review

## Abstract

**Background:**

The concept of neonatal near miss has been proposed as a tool for assessment of quality of care in neonates who suffered any life-threatening condition. However, there are no internationally agreed concepts or criteria for defining or identifying neonatal near miss. The purpose of this study was to perform a systematic review of studies and markers that are able to identify neonatal near miss cases and predict neonatal mortality.

**Methods:**

Electronic searches were performed in the Medline, Embase and Scielo databases, with no time or language restriction, until December 2014. The term “neonatal near miss” was used alone or in combination with terms related to neonatal morbidity/mortality and neonatal severity scores. Study selection criteria involved three steps: title, abstract and full text of the articles. Two researchers performed study selection and data extraction independently. Heterogeneity of study results did not permit the performance of meta-analysis.

**Results:**

Following the inclusion and exclusion criteria adopted, only four articles were selected. Preterm and perinatal asphyxia were used as near miss markers in all studies. Health indicators on neonatal morbidity and mortality were extracted or estimated. The neonatal near miss rate was 2.6 to 8 times higher than the neonatal mortality rate.

**Conclusions:**

Pragmatic and management criteria are used to help develop the neonatal near miss concept. The most severe cases are identified and mortality is predicted with these criteria. Furthermore, the near miss concept can be used as a tool for evaluating neonatal care. It is the first step in building management strategies to reduce mortality and long-term sequelae.

## Background

The Millennium Declaration promoted by the United Nations in 2000 included 8 Millennium Development Goals (MDG). These goals were expected to be achieved by the end of 2015 [1]. The fourth goal corresponds especifically to a reduction in mortality rate by two-thirds in children under 5 years of age, between 1990 and 2015. Despite major advances, some countries have still not reached this goal. It was estimated that 6.6 million children died in this age group in 2013 and 2.9 million deaths occurred in the neonatal period (0–27 days of life) [2].

The neonatal period is the most vulnerable time for infant survival, corresponding to almost 50 % of deaths occurring under 5 years of age. Identification and correction of factors that may improve maternal and neonatal care are likely to contribute to the reduction in mortality rates. From 1990 to 2013, 86 million deaths occurred in the neonatal period [2] and neonatal mortality dropped almost 40 % in the same period. The total number of deaths was 4.7 million in 1990 and 2.8 million in 2013. In the latter year, almost 1 million newborn infants died within the first day of life, corresponding to 16 % of all deaths in children under 5 years of age. In that same year, almost 2 million newborn children died within 7 days of life, representing 73 % of deaths in the entire neonatal period [2, 3].

The main causes of death in the neonatal period worldwide are complications arising from preterm birth, asphyxia during labor and sepsis, corresponding to ¾ of these deaths. It is known that the majority of neonatal deaths are preventable and the most effective way to reduce these deaths is to invest in maternal and neonatal care during childbirth and in the first 24 h after birth [1].

The odds of neonatal survival are dependent on family income, maternal school education and place of birth. Low-income, illiterate women and birth in rural zones decrease the odds of survival in the neonatal period. Children delivered in the urban zone into wealthy families, and born to more highly educated mothers still have a high death risk when they are delivered in low-income countries [2]. In Brazil, the infant mortality rate was 51 per 1000 live births in 1990, while it was 12 per 1000 live births (LB) in 2013. The goal for the end of 2015 is 20 deaths per 1000 LB. Neonatal mortality rate was 28 per 1000 LB in 1990 and 8 per 1000 LB in 2013 [4].

Despite a decrease in the neonatal mortality rate, particularly in wealthy, but also in low and middle-income countries, the morbidity rate remains elevated. It is estimated that the number of survivors from a “neonatal near miss” event is three to six times higher than the number of neonatal deaths. Therefore, in Brazil it is estimated that severe neonatal morbidity rates were at least fourfold higher when compared to the the mortality rate in 2011 [4]. However, there are still no definite criteria for morbidity of the neonatal population.

Many neonatal morbidity scoring systems exist. The question is whether these scoring systems can be used to define neonatal near miss. Systems as the Clinical Risk Index for Babies (CRIB) and SNAP (Score for Neonatal Acute Physiology) are used to evaluate neonatal care quality and individual prediction of death. The majority of existing scoring systems cannot be routinely applied to low-income and middle-income locations, due to their complexity and requirement of laboratory-based information. Furthermore, these systems are limited to certain newborn infant groups [5].

CRIB was created to predict the mortality rate of newborns under 32 weeks at birth and admitted to intensive care units. It cannot be used in term newborns [6, 7]. The greatest advantage of this system is its easy application and possibility of early scoring in the first 12 h of life, before neonatal care can interfere with scoring. In contrast, SNAP may be used at any gestational age. However, it has been criticized for having been developed from a cohort of few newborn infants weighing less than 1500 g (154 of the total number of 1643 newborn infants). Scores may be collected during the first 24 h of life, with several resources including each organ system and laboratory tests. Although the SNAP encompasses various organs and is good for predicting mortality, it is much more difficult to apply than the CRIB [5, 7, 8].

An optimal scoring system should be easy to use and apply early at the time of hospital admission. It must be reproducible, enabling the prediction of specific morbidity and mortality. It should also be applicable to all newborn infant groups [5]. Criteria selection used to compose this scoring system is of the utmost importance. There must be a balance between complex scoring systems with many variables that are difficult to execute, and a more simple system that is easy to use, but lacks accuracy [5].

The term near miss was originally imported from the aviation industry to the health sector. It was first employed to describe the occurrence of an unplanned event that did not result in injury, illness or damage, either by luck or appropriately applied interventions, i.e., the accident was prevented. With a systematized study of near miss accidents, centers that investigate and qualify airline services attempt to understand the chain of events leading to an accident and seek improvement.

In medical and public health terminology, maternal near miss (MNM) is the term for maternal morbidity in survivors of severe complications during pregnancy or the postpartum period. It has been estimated that severe maternal morbidity cases are several times more frequent than maternal death cases, and is a better indicator of healthcare quality. In countries with a low maternal mortality ratio, it would take many years to obtain a sufficiently large sample of maternal deaths for assessment of quality of maternal healthcare [9]. Uniformity in the definition of near miss cases could improve health care and develop an audit system. Unnoticed opportunities could be evaluated. Different centers and settings could also be compared [10].

Maternal near miss is defined by the World Health Organization as a woman who nearly died, but survived a complication during pregnancy, childbirth or within 42 days of termination of pregnancy. Operationally, it corresponds to any organ dysfunction or failure that threatens the life of a woman. It has become an important tool for a more thorough investigation of obstetric care. Simultaneously, it has contributed to the identification and diagnosis of women at risk and the initiation of early effective interventions, in addition to quality assessment of maternal health care [9]. However, despite the advantage of facilitating a common way to report this occurrence, maternal near miss criteria are still not universally accepted at the present time. Such criteria depend on contextual factors. In Malawi, Tanzania and the Netherlands, there is an ongoing process to optimize the standard WHO MNM criteria [11–13].

Similarly, neonatal near miss and neonatal mortality may also help identify deficiencies in neonatal care. However, there is currently no standard definition of neonatal near miss or any internationally agreed identification criteria for neonatal near miss cases. The term has been used in different contexts, such as any adverse event in intensive care units (encephalopathy, jaundice), or in sudden infant death syndrome and Brugrada Syndrome [14–17]. Used in a different context, in a manner similar to maternal near miss, it could contribute to the assessment and improvement of obstetric practice and perinatal care. A correlation could be established between neonatal near miss cases and neonatal deaths, aimed at decreasing adverse neonatal outcomes.

Analogous to the definition of maternal near miss, neonatal near miss would correspond to a morbid event that almost resulted in the death of an infant during the neonatal period, including criteria such as diseases, interventions and organ dysfunctions. Another definition proposed would be a newborn who nearly died, but survived a severe complication during birth or within the first 7 days of extrauterine life.

In South Africa, Mukuevho et al. [18] proposed a practical clinical definition of severe acute neonatal morbidity. It was applied in a pilot study in Kalafong in 2006, where the mortality rate was shown to be five times lower than the severe morbidity rate. Parameters assessed were dysfunction or failure of diverse body systems of the newborn until the third day of life: respiratory, cardiac, central nervous system, hypovolemia, hematologic, endocrine, renal, immunologic, musculoskeletal, and/or hepatic/gastrointestinal. Parameters were similar to those used to define maternal near miss.

The aim of this study was to conduct a comprehensive systematic review on neonatal near miss, searching for studies in the scientific literature that analyzed neonatal morbidity markers as criteria for identifying and defining neonatal near miss. In addition, results of scientific article related to studies that used these markers to predict severe neonatal morbidity (classified as ‘neonatal near miss’) and neonatal deaths were identified and compiled.

## Method

A systematic review was carried out, following instructions from the PRISMA guidelines for systematic reviews [19]. Medline, Embase and Scielo electronic databases were accessed, and a systematic search was carried out, using the keyword “neonatal near miss”, alone or with a combination of keywords: (neonatal morbidity) AND (neonatal illness severity score) OR (neonatal disease severity score) AND (neonatal mortality). Only studies that had a clear definition of neonatal near miss, established criteria and that contained original data were considered eligible. There were no time (until December 2014) or original language restrictions.

Refinement of the search took place after the initial search was made. Articles of interest were selected in three steps. The first seletion was based on article title. The second was based on article abstract and the third on the full text article. In addition, the reference lists of identified studies were also evaluated, in an attempt to find other eligible studies. Two researchers independently participated in the process of data search selection and extraction. The results between both researchers were compared to check for any possible disagreement, which was solved by the opinion of a third senior researcher.

Inclusion criteria were studies conducted in the neonatal period, within 28 days of life, with newborns over 22 weeks of gestational age and weight greater than 500 g. Exclusion criteria were the absence of any definition of neonatal near miss or report of only neonatal mortality as the outcome.

In the studies included, emphasis was placed on differences in definitions and criteria for identification of neonatal near miss cases. Study results were qualitatively compiled, including differential evaluation of the main causes of neonatal morbidity/mortality, use of pragmatic criteria for severe morbidity (birth weight, gestational age, Apgar score, etc.) and management criteria for severity as possible markers for predicting neonatal death. The nature and heterogeneity of data from each selected study did not enable us to carry out a meta-analysis of the results. Data were compiled using a qualitative approach. To combine selected study results, we reported the number of corresponding live births, variables used as neonatal near miss criteria, specific neonatal period of data collection, neonatal mortality rate, neonatal near miss rate, neonatal mortality index and severe neonatal outcomes rate [20–23] for each study. Contrary to findings in maternal morbidity studies [9], the indicators used for neonatal morbidity are actual rates and not ratios.

Some figures presented were not originally available in the correspondent article and were obtained from published numbers or direct contact with the authors for further information.

## Results

**Fig. 1:**
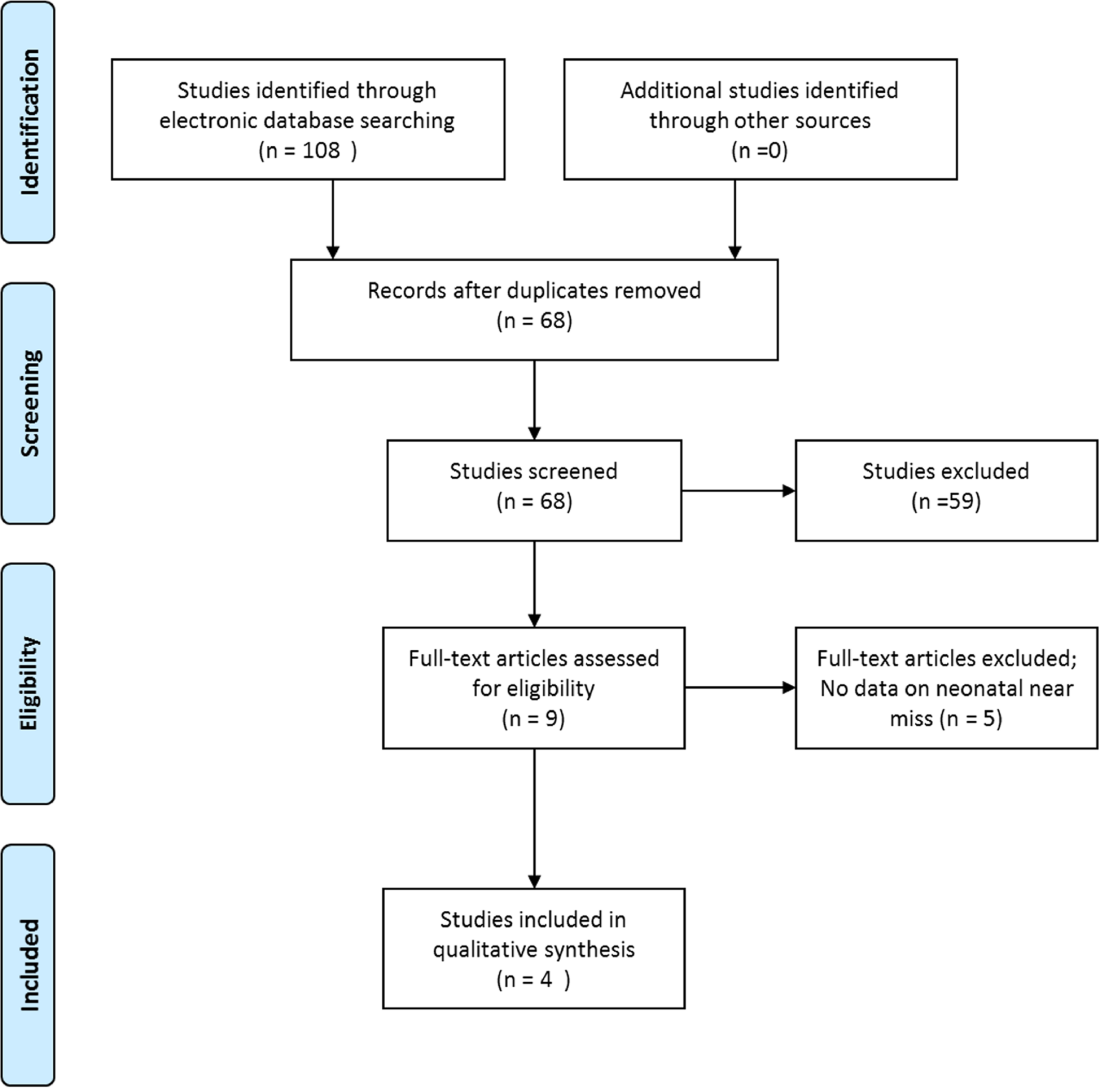
Flowchart of study search and inclusion in the review

The electronic search resulted in 41 articles in Medline, 61 articles in Embase and 6 articles in Scielo (total of 108 articles). Forty (40) duplicates were observed and a total of 68 titles were analyzed. After the abstracts were read, 9 articles still remained in the selection. Finallly, after full-text reading of the articles, only 4 studies were considered eligible for the current systematic review (Fig. 1). Of the 5 articles excluded after full text reading, one article did not show numerical data of near miss cases and the remainder demonstrated data related only to neonatal mortality outcome. All selected review articles varied widely in terms of concept and criteria used. Therefore, planning to undertake a meta-analysis was considered inappropriate. Thus, study results were briefly presented individually and were subsequently compiled in a qualitative manner:

**Study 1**: Avenant et al. [20] (Table 1)

**Table 1:** Comparison of proportions of main causes between neonatal near miss (NNM) and neonatal deaths (NND), South Africa (Modified from Avenant et al., 2009) [17]

Primary cause of morbidity or death	NNM (%)	NND (%)	Total
Intrapartum asphyxia	12.9	4.2	11.1
Birth trauma	10.8	-	8.5
Antepartum hemorrhage	14	4.2	12
Hypertension	8.6	12.5	9.4
Spontaneous preterm birth, premature rupture of			
membranes, multiple pregnancy	40	62.5	44.4
Congenital malformation	-	8.3	1.7
Maternal Infection	1.1	4.2	1.7
Unknown	12.9	4.2	11.1

This study included 3770 liveborn infants, using data from “Saving Babies: 2003–2005: Fifth Perinatal Care Survey of South Africa.” The primary obstetric causes of early neonatal death were initially listed and related to neonatal near miss.

To define neonatal near miss, Mukwevo [18] suggested the use and application of markers until 3 days of life. An infant mortality rate of 6.3/1000 live births and a neonatal near miss rate of 24.7/1000 LB were shown. The majority of neonatal near miss cases were caused by respiratory dysfunction/failure (63 %) followed by immunologic dysfunction/ failure, including infections (21.2 %), and subsequently central nervous system dysfunction/failure (5 %). Compared to the neonatal mortality rate, more cases of neonatal near miss were observed among obstetric cases in which asphyxia, trauma or antepartum hemorrhage had occurred.

**Study 2**: Pileggi et al. [21] (Table 2)

**Table 2:** Performance of life-threatening neonatal conditions with their 95 % confidence intervals, Brazil, WHO (Modified from Pileggi et al., 2010) [18]

Condition	Early neonatal deaths			Sensitivity	Specificity	Positive Likelihood Ratio
		+	−	a/(a + c)	d/(b + d)	Sensitivity/1-Specificity
	+	a	b			
	-	c	d			
Gestational age at birth < 30 weeks	+ -	52 64	69 14.618	44.8 % (36.1-53.9)	99.5 % (99.4-99.6)	95.4 (70.0-130.1)
Very low birthweight	+ -	74 50	138 14.863	59.7 % (50.9-67.9)	99,1 % (98.5-99.2)	64.9 (52.0-80.9)
5 min Apgar score < 7	+ -	66 50	194 14.630	56.9 % (47.8-65.5)	98.7 % (98.5-98.9)	43.5 (35.2-53.7)
Any of the above conditions	+ -	100 21	324 14.175	82.6 % (74.9-98.0)	97.8 % (97.5-98.0)	37.0 (32.3-42.3)

In this study, a secondary analysis of the Brazilian database on the “2005 WHO Global Survey on Maternal and Perinatal Health” was carried out, including 15169 live births. Live birth records with vital status known on the 7^th^ day of life or at hospital discharge were included.

A definition of neonatal near miss was developed based on the major causes of death: preterm birth and perinatal asphyxia. Risk factors were: low birthweight, less than 30 weeks of gestational age at birth, and Apgar score < 7 at 5 min of life. The early neonatal mortality rate, neonatal near miss rate, in addition to the Severe Neonatal Outcomes Rate (number of deaths in the first week of life added to the number of neonatal near miss cases per 1000LB) and neonatal mortality index (number of deaths in the first week of life among those presenting neonatal severe outcomes) were analyzed. The early neonatal mortality rate was 8.2 /1000LB and the neonatal near miss rate was 21.4 /1000LB.

**Study 3**: Pileggi-Castro et al. [22] (Table 3)

**Table 3:** Frequency of markers of severity among live born neonates (*n* = 309 644). Pragmatic markers (any pragmatic marker of severity), WHO ^a^ (Modified from Pileggi-Castro et al., 2014) [19]

Markers of severity	Severe neonatal outcomes ^b^	Neonatal Near Miss	Early neonatal death	Mortality Index
	*n* (‰)	*n* (‰)	*n* (‰)	%
Pragmatic markers of severity				
Apgar score <7 at 5^th^ minute	8033 (25.9)	6745 (21.8)	1288 (4.2)	16.0
Birthweight <1750 g	6099 (19.7)	4456 (14.4)	1643 (5.3)	26.9
Gestational age <33 weeks	4438 (14.3)	3424 (11.1)	1014 (3.3)	22.8
Any pragmatic marker of severity	13 795 (44.6)	11 587 (37.4)	2208 (7.1)	16.0
Management markers of severity				
Use of intravenous antibiotics	13 496 (43.6)	11 952 (38.6)	1544 (5.0)	11.4
Nasal CPAP	4772 (15.4)	3874 (12.5)	898 (2.9)	18.8
Any intubation (in the first 7 days)	3970 (12.8)	2811 (9.1)	1159 (3.7)	29.2
Use of phototherapy in the first 24 h	3434 (11.1)	3222 (10.4)	212 (0.7)	6.2
Cardio pulmonary resuscitation	2961 (9.6)	1598 (5.2)	1363 (4.4)	46.0
Use of any vasoactive drug	1890 (6.1)	1176 (3.8)	714 (2.3)	37.8
Use of anticonvulsants	1441 (4.7)	1166 (3.8)	275 (0.9)	19.1
Use of surfactant	1366 (4.4)	1075 (3.5)	291 (0.9)	21.3
Transfusion of blood derivatives	980 (3.2)	802 (2.6)	178 (0.6)	18.2
Use of corticosteroid for treatment of refractory hypoglycemia	895 (2.9)	736 (2.4)	159 (0.5)	17.8
Any surgical procedure	247 (0.8)	216 (0.7)	31 (0.1)	12.6
Any management marker of severity	18 673 (60.3)	16 421 (53.0)	2252 (7.3)	12.1
Combined markers (any pragmatic or management marker)	25 103 (81.1)	22 458 (72.5)	2645 (8.5)	10.5

^a^Calculated using the dataset of the WHO Multicountry Survey on Maternal and Newborn Health (2010–2011)

^b^Calculated by the sum of neonatal near-miss cases and early neonatal deaths

In this study, secondary analyses of two WHO databases were carried out: the “Global Survey on Maternal and Perinatal Health” (WHOGS) with 277,706 live births and the “Multicountry Survey on Maternal and Newborn Health” (WHOMCS) with 309,644 live births. Live birth records were included with vital status known on the 7^th^ day of life or at hospital discharge.

In the WHOGS database, pragmatic neonatal near miss criteria were developed using three conditions associated with preterm and perinatal conditions: low birthweight (<1750 g), gestational age under 33 weeks at birth and Apgar score <7 at 5 min of life. These three variables were considered pragmatic markers for the prediction of early neonatal death and were subsequently used in the WHOMCS database.

WHOMCS reported specific data on management of severe neonatal morbidity, in addition to the three previously reported variables. The following management markers of severity were based on interventions used in a South African study: use of intravenous antibiotics, use of nasal CPAP or intubation at any time point in the first week of life, cardiopulmonary resuscitation, use of any vasoactive drug, use of phototherapy in the first 24 h of life, use of anticonvulsants, administration of surfactant, use of blood transfusion, use of corticosteroids in refractory hypoglycemia, and any surgical intervention performed in the first week of life.

Studied indicators were modified and adapted similarly to the WHO indicators of maternal near miss. However, rates were used rather than ratios. The study determined the early neonatal mortality rate, neonatal near miss rate, severe neonatal outcomes rate, the ratio between neonatal near miss and each neonatal death, and the neonatal mortality index.

The overall early neonatal mortality rate was 9.2/1000 LB and neonatal near miss rate was 72.5/1000 LB considering any pragmatic or management marker. For pragmatic markers, the neonatal near miss rate was 37.4/1000 LB and for management markers, the neonatal near miss rate was 53/1000 LB.

**Study 4**: Silva et al. [23] (Table 4)

**Table 4:** Risk factors for neonatal death by adjusted analysis, Birth in Brazil survey, 2011–2012 (Modified from Silva et al., 2014) [20]

Variables	*n* (unweight)^a^	By 1000 (weighted)	Odds ratio (95 %CI)^b^	*p*-value*
Birthweight (g)				
≥ 2500	21740	2.2	1	
1500 to 2499	1763	31.3	5.38 (1.83–15.84)	<0.001
< 1500	321	407.9	10.51 (3.00–36.83)	<0.001
Apgar score at 5^th^ minute				
≥ 7	22909	7.1	1	
< 7	211	369.8	15.98 (6.02–42.38)	<0.001
Mechanical ventilation				
No	23631	3.1	1.00	
Yes	430	370.7	14.47 (6.90–30.35)	<0.001
Gestational age (weeks)				
≥ 37	21174	2.2	1.00	
32–36	2092	20.6	1.30 (0.47–3.62)	0.641
< 32	336	386,3	5.13 (1.59–16.52)	0.006
Congenital malformation				
No	23914	9.5	1.00	
Yes	147	230.3	15.50 (5.88–40.87)	<0.001

^a^numbers may not add up to total (24061) because of missing values

^b^Odds ratio calculated from multiple logistic regression with adjustment for all variables on the table

*P-value calculated by the log-likelihood ratio

This study included 24,061 live births from the the “Birth in Brazil” survey database. Variables used were Apgar score < 7 at 5 min of life, gestational age (≤32, 33 to 36 and ≥ 37 weeks), birthweight (<1500, 1500 to 2499 and ≥2500 g), multiple births, use of mechanical ventilation, use of supplemental oxygen after birth, neonatal intensive care admission, use of nasal CPAP, tracheal intubation in the delivery room, cardiac massage, resuscitation drugs, phototherapy in the first 72 h of life, use of surfactant, use of antibiotics in the first 48 h of life, congenital malformation, seizures, respiratory diseases of the newborn (transient tachypnea, hyaline membrane disease, pulmonary hypertension or meconium aspiration syndrome), hypoglycemia or necrotizing enterocolitis.

Odds ratios were calculated to estimate the association between selected factors and neonatal death. All newborn infants who survived the neonatal period, and had at least one of the variables chosen were considered neonatal near miss cases. Sensitivity, specificity, positive and negative predictive values were used, in addition to the log-likelihood ratios to evaluate the power of each indicator of neonatal near miss.

Neonatal mortality rate was 11.1/1000 LB and neonatal near miss rate was 39.2/1000 LB. Variables associated with neonatal death were birthweight < 1500 g, Apgar score <7 at 5 min of life, use of mechanical ventilation, preterm infants < 32 weeks and newborns with congenital malformations. These variables were chosen as indicators of neonatal near miss.

Data from the four studies were compiled in a table for comparison of characteristics and corresponding indicators of Neonatal Mortality Rate, Neonatal Near Miss Rate, Neonatal Mortality Index and Severe Neonatal Outcomes Rate (Table 5). Generally speaking, the Neonatal Near Miss Rate was higher in studies that combined pragmatic and management markers of severity as criteria for near miss, the same occurring with the Severe Neonatal Outcomes Rate. As expected, the neonatal mortality rate was higher in studies including longer neonatal periods. In addition, studies using more comprehensive criteria for neonatal near miss had the lowest neonatal mortality indices.

**Table 5:** Comparison of characteristics and results of studies included in the review

Author	Year	Number of Live Births	Variables as criteria for neonatal near miss	Neonatal period (days)	Neonatal mortality Rate (/1000LB)	Neonatal Near Miss Rate (/1000LB)	Neonatal Mortality Index (%)	Severe Neonatal Outcomes Rate (/1000 LB)
Avenant	2009	3770	Criteria of Mukwevo	Up to 3 days	6.3^a^	24.7	20.5	31.0
Pileggi	2010	15169	Birthweight < 1500 g Apgar < 7 at 5^th^ min Gestational age < 30 weeks	Up to 7 days	8.2^b^	21.4	27.7	29.5
Pileggi-Castro WHOGS	2014	277706	Birthweight < 1750 g Apgar < 7 at 5^th^ min Gestational age < 33 weeks	Up to 7 days	7.4^b^	44.4	14.2	51.8
Pileggi-Castro WHOMCS	2014	309644	Pragmatic markers	Up to 7 days	9.2^b^	37.4	19.7	46.6
			Management markers		9.2^b^	53.0^a^	14.7	62.2
			Combined markers		9.2^b^	72.5	12.7	81.7
Silva AA	2014	24061	Birthweight < 1500 g Apgar < 7 at 5^th^ min Gestational age <32 weeks Congenital malformation Mechanical ventilation	Up to 28 days	11.1	39.2	22.1	50.3

^a^ Under 3 days neonatal mortality rate; ^b^Early neonatal mortality

## Discussion

Criteria for preterm birth and perinatal asphyxia, major causes of neonatal death, were used in all studies to help develop a pragmatic definition of near miss [20–23]. The neonatal period included in each study ranged from 3 to 28 days of life. In two studies, newborn infants were evaluated in the early neonatal period. Only one study emcompassed the entire neonatal period up to 28 days. Although different criteria and markers were adopted for evaluation of neonatal near miss, all studies analyzed showed that the neonatal near miss rate was 2.6 to 8 times higher than the neonatal mortality rate (case fatality ratio for neonatal near miss). Taking only neonatal mortality into account, many cases of severe neonatal morbidity may not be analyzed. As a result, a limited understanding of the determinants and factors associated with poor neonatal performance may occur. One reason why metanalysis could not be conducted in this review was the heterogeneous nature of the studies.

The neonatal mortality rate was relatively similar among studies (6.3 to 11.1 per 1000LB). The highest rate occurred in the Brazilian study covering the full neonatal period [23]. In the largest WHO study [22], more comprehensive criteria such as gestational age (<33 weeks) and weight superior to those in other studies (<1750 g) were considered. This was probably due to the higher proportion of low-income and middle-income countries contributing to the sample, resulting in worse maternal and neonatal conditions. The set of criteria became less sensitive and higher proportion of more severe cases were selected, increasing the neonatal near miss rate.

The neonatal near miss rate was higher in studies that evaluated pragmatic criteria combined with management criteria. Every newborn who had any of these criteria was considered a near miss case, and more than 70 neonatal near miss cases per thousand live births were reported in the WHO Muticountry Survey [22]. In contrast, the mortality index was lower in the study combining pragmatic and management criteria, as expected. The association between pragmatic and management criteria most probably permitted the evaluation of a larger number of surviving newborns considered to be at risk. It is important to highlight that neonatal near miss criteria are unable to identify the total number of neonatal deaths, using either pragmatic, management or a combination of these criteria at birth. A small proportion of cases, including sudden neonatal death, congenital malformation, or late neonatal sepsis or meningitis, not identified as a neonatal near miss events at birth. This may be confirmed by the neonatal death detection rate of only 93 % in the WHO study [22].

Although congenital malformation performed well as a marker of severity, it is noteworthy that many deaths resulting from these malformations may not have been prevented even with effective interventions. Quality assessment of health care thus may not have been performed properly. In fact, it appeared in only one selected study [23]. The same question arises regarding extremely premature infants and a better assessment should be made. These issues should be more fully addressed in high-income settings, where technical and financial resources are more widely available and there is no urgent need to prioritize areas of investment.

This study clearly has some limitations. It was an initial attempt to perform a systematic review on a relatively new topic There is a lack of detailed studies on the concept, criteria and occurrence. Some studies may not have been identified simply because they still had not been published or the search terms used failed to select these studies. Computerized data refer only to infants delivered inside a hospital. It was not possible to evaluate the relationship between severe morbidity and mortality in newborns born outside a hospital. This could be of interest, at least in settings with a higher proportion of deliveries at home or in the community.

Studies were conducted in countries with huge disparities in socioeconomic conditions, which might contribute to cases of severe morbidity and neonatal mortality [20, 21, 23]. South Africa has a medium HDI (Human Development Index) and Brazil has a high HDI, making a comparison between studies difficult. The only study showing global data from countries with different socioeconomic conditions was the WHO study in its two components [22]. However, the number of newborns from countries with a very high HDI was only 10 %. It was not possible to assert that the results could be fully generalized. Management markers may be more important to countries with low mortality rates, since these markers are more subtle than death and perform more effectively than pragmatic markers, implying in better health care conditions. It may not be possible to use any neonatal near miss criteria for cross-country comparisons, since supplementary management criteria (CPAP, use of surfactant, etc.) are clearly context-dependent, unless settings or health facilities from the same level of complexity are compared.

Reviewed studies were mainly retrospective analyses of perinatal care and outcomes. It is crucial to perform a large prospective study that is designed to obtain corroborating data to construct a concept of neonatal near miss. Studies that investigated the long-term consequences of neonatal near miss events and not only those occurring in the first month of life could also be useful. Thus, it is important to improve the concept of neonatal near miss to predict future developmental problems related to high-risk conditions of earlier infant exposure.

A combination of pragmatic markers and management markers of severity identified a higher number of near miss cases. This combination seems to perform well as a predictor of early neonatal death and can identify more than 90 % of these deaths [22]. Validation of a neonatal near miss concept, as well as indicators for its application could be useful for the exploration of health care quality worldwide. Furthermore, priorities could be established in the management of these newborn infants, improving neonatal health care and thus decreasing the negative impact on the future lives of these children.

## Conclusions

Improvement, concordance and validation of a simple, easy and standard definition of neonatal near miss is required. To define the term, criteria should be simple, feasible to use in individual health care facilities and at the health system level. It should also be meaningful to clinicians, managers and health care professionals. It needs to be stable in terms of severity and applicable to a variety of settings, regardless of the local development level. From the currently available results, the use of the pragmatic criteria for neonatal near miss is recommended, whenever possible. For this purpose, the three criteria identified and included by the largest WHO study (Apgar <7, birthweight < 1750 g and gestational age <33 weeks) should be employed. The three criteria are part of the vital health indicators routinely collected and may be retrospectively estimated. For a more detailed prospective evaluation, in locations with more substantial resources, the combination of the 3 criteria with managment criteria for severity (indicating dysfunction or failure of organs and systems) appears to be the best option available for the identification of neonatal near miss cases.

At institutions or settings where there are low neonatal mortality rates, near miss cases may be assessed as supplemental resources to evaluate health care services and identify issues of health care quality. The major problem of neonatal near miss currently lies in its definition. Another issue is how to carry out an audit of existing services. The PAHO (Pan American Health Organization) has played a role in this regard by supporting a meeting of experts in the field with the common purpose of reaching a uniform definition, proposing standard criteria for use in different settings and proposing a pilot prospective surveillance system for validation of the concept and criteria of near miss [24]. There is an urgent need to standardize the near miss concept and criteria, preferably by an international organization such as the WHO, for comparisons among different contexts and hopefully elaboration of a package of recommended interventions for each specific severe neonatal morbidity condition identified.

## Abbreviations

CRIB: Clinical risk index for babies
GS: Global survey
HDI: Human development index
LB: live births
MCS: Multi- country survey
MDG: Millennium development goals
MNM: Maternal near miss
NMI: Neonatal mortality index
NMR: Neonatal mortality rate
NNMR: Neonatal near miss rate
NNM: Neonatal near miss
SNAP: Score for neonatal acute physiology
SNOR: Severe neonatal outcomes rate
WHO: World Health Organizaton
